# A case report of osilodrostat treatment in primary bilateral macronodular adrenal hyperplasia associated with bilateral adrenal mass reduction

**DOI:** 10.1186/s13256-026-06442-9

**Published:** 2026-08-03

**Authors:** Wenwei Xie, Wenqiong Xu

**Affiliations:** 1 Department of Endocrinology and Metabolism, First Affiliated Hospital of Nanchang University, Nanchang, 330006 Jiangxi China; 2 Jiangxi Clinical Research Center for Endocrine and Metabolic Disease, Nanchang, China; 3 Jiangxi Branch of National Clinical Research Center for Metabolic Disease, Nanchang, China

**Keywords:** Primary bilateral macronodular adrenal hyperplasia, Osilodrostat

## Abstract

Primary bilateral macronodular adrenal hyperplasia (PBMAH) is a rare cause of Cushing syndrome (CS), characterized by bilateral adrenal nodules larger than 1 cm. Cortisol hypersecretion in PBMAH is driven by intra-adrenal rather than pituitary adrenocorticotropic hormone (ACTH), with its pathogenesis mediated by aberrant hormone receptor expression in the adrenal cortex. This article reports a case of a 60-year-old woman with PBMAH in whom a marked reduction in bilateral adrenal masses was observed following treatment with osilodrostat. The patient presented with typical Cushingoid features, accompanied by hypertension, diabetes mellitus, osteoporosis, and severe restrictive lung disease. Genetic testing identified a pathogenic mutation in the ARMC5 gene, confirming the diagnosis. Because she was intolerant of surgical treatment, left adrenal artery embolization (AAE) was performed but failed to control hypercortisolism. Osilodrostat therapy was subsequently initiated—this potent 11β-hydroxylase inhibitor was recently approved in China for the treatment of Cushing syndrome. Notably, after 23 weeks of treatment, the patient's 24-hour urinary free cortisol (24 hours-UFC, CLIA) level returned to normal. Treatment was then discontinued, and during 11 months of follow-up the patient’s 24 hours-UFC remained within the normal range. Moreover, imaging performed during the period of osilodrostat therapy demonstrated significant shrinkage of both adrenal glands. To our knowledge, this is the first reported case of bilateral adrenal mass reduction observed in temporal association with osilodrostat treatment in a PBMAH patient. However, prior AAE confounds the attribution of tumor shrinkage to osilodrostat alone. These findings highlight an observed association between the drug and mass reduction, warranting further investigation to clarify its potential role beyond symptomatic control.

## Introduction

Primary bilateral macronodular adrenal hyperplasia (PBMAH) is a distinct subtype of Cushing's syndrome (CS) in which cortisol hypersecretion is driven by intra-adrenal rather than pituitary ACTH, with its pathogenesis driven by aberrant hormone receptor expression in the adrenal cortex. PBMAH accounts for less than 2% of confirmed CS cases [1]. Osilodrostat is a potent 11β-hydroxylase (CYP11B1) inhibitor for the treatment of adult CS. This article reports a case of PBMAH in which bilateral adrenal masses were reduced following treatment with osilodrostat. To date, no published reports have described the use of osilodrostat in PBMAH, making this the first documented case and providing clinical insight for therapeutic decision-making.

## Case presentation

A 60-year-old woman presented to our hospital with hypertension, hyperglycemia, chest tightness, weight gain, back pain, and decreased physical activity capacity. Her height was 149.0 cm, her weight was 71.0 kg, and her body mass index (BMI) was 31.98 kg/m^2^. Her vital signs were as follows: blood pressure of 149/75 mmHg. She had obvious Cushingoid features: full moon face, buffalo hump, thin skin, and central obesity. Family history: her brother had a history of Cushing's syndrome.

### Diagnostic assessment

**Fig. 1:**
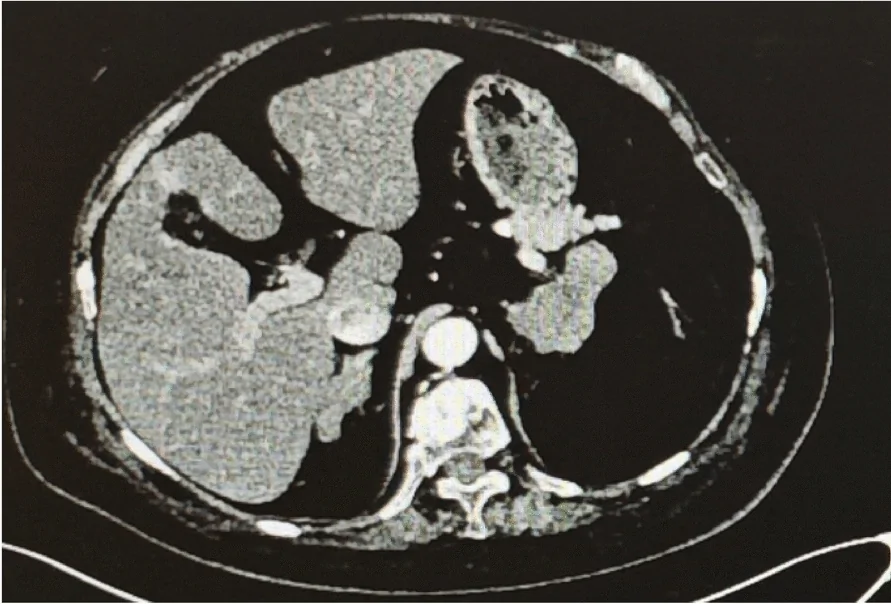
Adrenal computed tomography findings of the patient. Note: Bilateral adrenal glands show diffuse heterogeneous low-density nodules of varying sizes, with indistinct margins and partial confluence. The maximum cross-sectional diameters of the left adrenal gland are 58.21 mm × 33.40 mm, and those of the right adrenal gland are 44.65 mm × 18.20 mm. The estimated maximum cross-sectional areas (product of orthogonal diameters) were 1944.6 mm^2^ (left) and 812.6 mm^2^ (right)

Laboratory examinations are shown in Tables 1 and 2. Chest and adrenal computed tomography (CT) revealed: scattered chronic infectious lesions in both lungs; multiple bilateral rib fractures with callus formation; partial thoracic vertebral compression fractures; severe hepatic steatosis; and bilateral adrenal diffuse enlargement with multiple low-density nodules, suggestive of adenoma-like hyperplasia (Fig. 1). Lumbar magnetic resonance imaging (MRI) showed flattening of the T12–L5 vertebral bodies, indicating compression fractures. Pituitary MRI demonstrated slightly decreased enhancement in the posterior pituitary. Bone mineral density indicated osteoporosis. Pulmonary function testing revealed severe restrictive ventilatory dysfunction. Genetic testing (method: targeted region capture and high-throughput sequencing) in the patient and her brother identified a pathogenic or likely pathogenic variant consistent with the clinical presentation, located in the armadillo repeat containing 5 (ARMC5) gene, suggestive of PBMAH type 2. Based on medical history and auxiliary examinations, the diagnosis was: primary bilateral macronodular adrenal hyperplasia (PBMAH); secondary diabetes mellitus; secondary hypertension; severe osteoporosis; severe hepatic steatosis; and severe restrictive ventilatory dysfunction.

**Table 1:** Laboratory data on administration

Blood test	Results	Reference ranges
Red blood cell	4.2	3.8–5.1 × 10^12/L
White blood cell	6.33	3.5–9.5 × 10^9/L
Differential count		
Neutrophils	83.30%	
Lymphocytes	9.00%	
Eosinophils	0.50%	
Blood glucose	**10.0**	3.9–6.1 mmol/L
HbA_1c_	**8.60%**	4–6%
C-peptide	**4.43**	0.1–4.4 ng/mL
LH	37.90	≤ 58.5 mIU/mL
FSH	57.50	≤ 134.8 mIU/mL
E2	16.20	5.0–138 pg/mL
T	8.88	2.9–40.8 ng/dL
PRL	15.90	4.79–23.3 ng/mL
P	**0.37**	0.05–0.126 ng/mL
DHEA-S	**14.74**	18.9–205 μg/dL
GH	**0.05**	0.126–9.88 ng/mL
IGF-1	140.260	85–230 ng/mL
FT3	**2.27**	2.3–4.2 pg/mL
FT4	**0.88**	0.93–1.7 ng/dL
TSH	2.550	0.27–4.2 μIU/mL
TPOAb	13.5	0–115 IU/mL
TGAb	10.6	0–34 IU/mL
PTH	57.8	15–65 pg/mL
Aldosterone	70.55	10–160 pg/mL
Renin	17.49	2.4–32.8 pg/mL
ARR	4.03	0–57
Cortisol rhythm	**31.07(8AM)**	4.26–24.85 μg/dL
	**28.08(4PM)**	2.9–17.3 μg/dL
	**17.02(12MN)**	2.9–17.3 μg/dL
ACTH	**2.81**	7.2–63.4 pg/mL
24 hours-UFC	**2484**	19.3–317.5 μg/24 hours,CLIA
Creatinine	**33.0**	41–81 μmol/L
BUN	3.2	3.1–8.8 mmol/L
eGFRCre	115.1	> 90 mL/minute/1.73m^2^
Sodium	**147.5**	137–147 mmol/L
Calcium	**1.93**	2.11–2.52 mmol/L
Chloride	110	99–110 mmol/L
Potassium	**3.33**	3.5–5.3 mmol/L

Abnormal values are shown in bold font. Values are International System of Units (SI)

ACTH, adrenocorticotropin; UFC, urinary free cortisol; BUN, blood urea nitrogen; FSH, follicle-stimulating hormone; LH, luteinizing hormone; PRL, prolactin; E2, estradiol; P, progesterone; T, testosterone; DHEA-S, dehydroepiandrosterone sulfate; eGFRCre, estimated glomerular filtration rate from creatinine; HbA_1c_, glycated hemoglobin A_1c_; TSH, thyroid stimulating hormone; FT3, free triiodothyronine; FT4, free thyroxine; TPOAb, thyroid peroxidase antibody; TGAb, thyroglobulin antibody; PTH, parathyroid hormone; IGF-1, insulin-like growth factor 1; GH, growth hormone

**Table 2:** Low-dose dexamethasone suppression tests (LDDST) and high-dose dexamethasone suppression tests (HDDST)

Time	Morning serum cortisol (Μg/dl)	ACTH (pg/ml)	24 hours-UFC (Μg/24 hours)
Pre-LDDST baseline	36.06	2.62	-
1 day after LDDST	30.47	2.53	1528.6
2 days after LDDST	27.91	< 1.5	1866.2
Pre-HDDST baseline	63.99	2.35	2484
1 day after HDDST	39.65	2.59	2960
2 days after HDDST	49.09	2.53	2395

### Treatment

Treatment included insulin for glycemic control; nifedipine controlled-release tablets, sacubitril/valsartan, bisoprolol fumarate, and spironolactone for blood pressure control; alongside anti-infective therapy, anti-osteoporotic treatment, and respiratory support. After stabilization of blood glucose and blood pressure, left adrenalectomy was planned (imaging showed significantly larger hyperplastic tissue on the left side), but the patient could not tolerate surgery. Therefore, on September 30, 2024, left adrenal artery embolization (AAE, also known as adrenal artery ablation in China) was performed.

### Outcome and follow-up

**Fig. 2:**
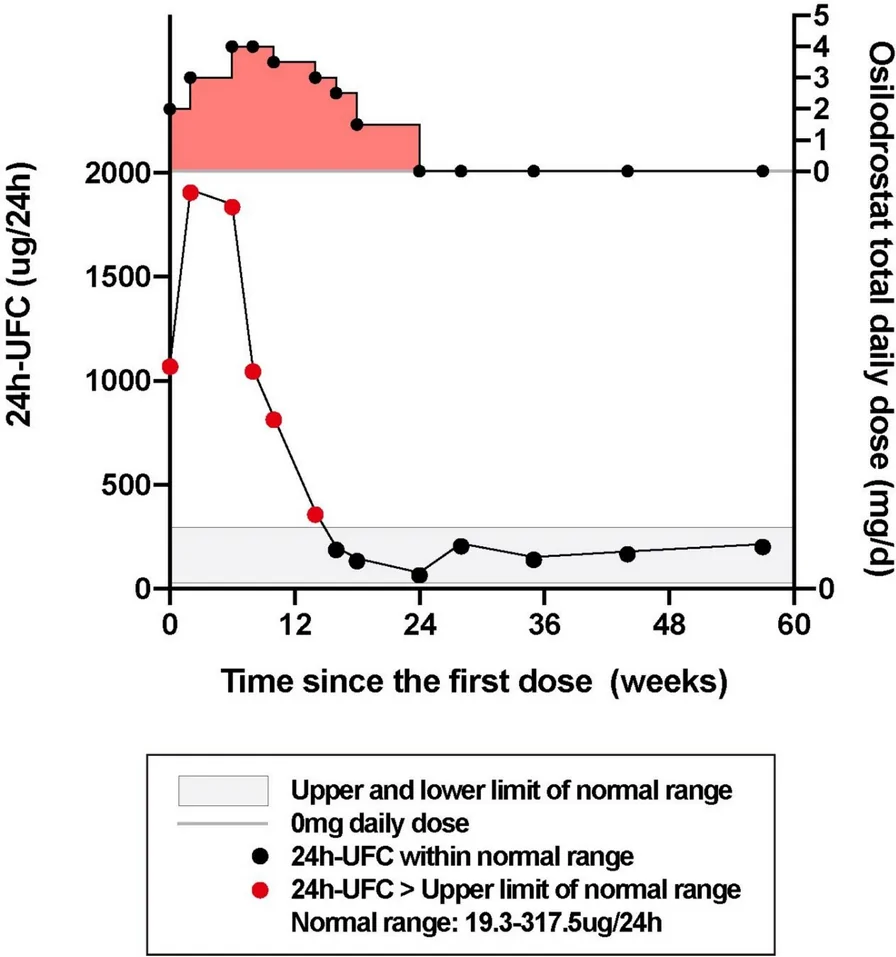
Changes in 24 hours-UFC and corresponding dose adjustments during osilodrostat therapy

**Fig. 3:**
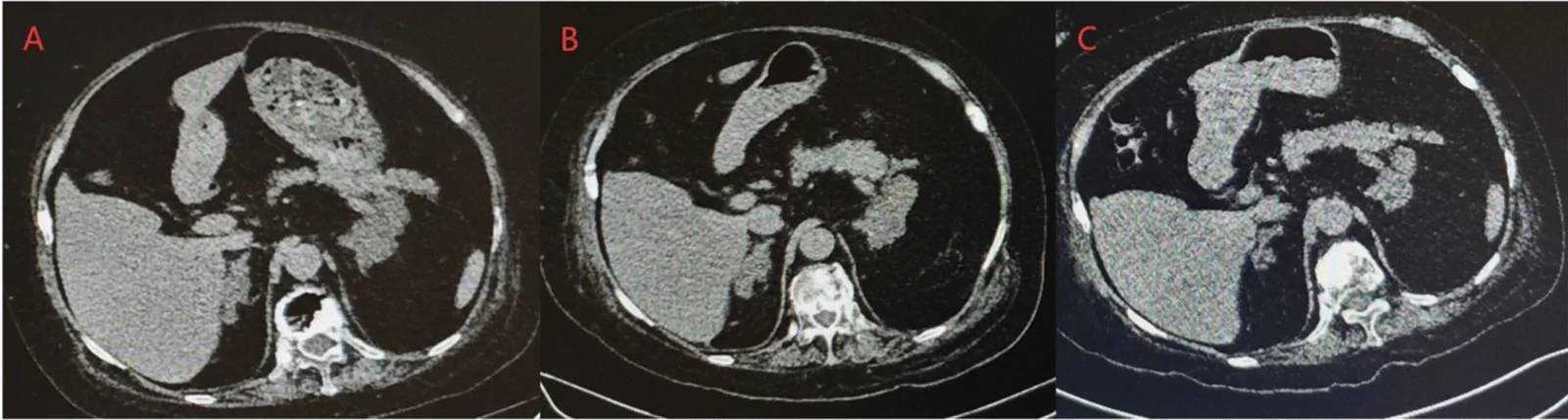
Serial adrenal computed tomography follow-up after left adrenal artery embolization (AAE) and osilodrostat therapy. **A** 1 month after left AAE. **B** 3 months after left AAE and 1 month after initiation of osilodrostat. C: 8 months after left AAE and 5 months after initiation of osilodrostat. Note: Low-density nodules in the left adrenal gland progressively decreased in size, with clearer margins. The maximum cross-sectional diameters were 55.51 mm × 30.70 mm, 53.57 mm × 28.07 mm, and 42.32 mm × 24.57 mm, respectively. The estimated maximum cross-sectional areas (product of orthogonal diameters) were 1704.2 mm^2^, 1503.7 mm^2^, and 1039.8 mm^2^. Low-density nodules in the right adrenal gland also gradually decreased in size, with maximum cross-sectional diameters of 47.22 mm × 19.92 mm, 38.76 mm × 16.39 mm, and 35.86 mm × 14.41 mm. The estimated maximum cross-sectional areas were 940.6 mm^2^, 635.3 mm^2^, and 516.7 mm^2^

Postoperatively, hypercortisolism persisted. Osilodrostat treatment was initiated on December 9, 2024, starting at 2 mg/day, with dose adjustments every two weeks (Fig. 2, Table 3). Considering prior left AAE, adrenal CT was monitored after treatment (Fig. 3). After 6 weeks of osilodrostat therapy, at a dose of 4 mg/day, a marked downward trend in 24-hour urinary free cortisol (24 hours-UFC) was observed. The dose was then gradually reduced, reaching 1 mg/day by treatment week 22, while 24 hours-UFC continued to decline. At the time, the cortisol rhythm was: 6.16 (8 AM)–6.90 (4 PM)–6.09 (12 NM). Subsequently, symptoms of hypocortisolism appeared, including nausea and decreased appetite. Therefore, osilodrostat was discontinued after 23 weeks of treatment. The patient continued to experience nausea and anorexia after discontinuation. On May 23, 2025, morning serum cortisol was 3.91 μg/dL, and hydrocortisone replacement therapy (10 mg at 08:00 and 5 mg at 16:00) was initiated. After one week, symptoms improved, morning serum cortisol increased to 20.97 μg/dL, and hydrocortisone was stopped. Osilodrostat remained discontinued thereafter, with repeated 24 hours-UFC measurements consistently within the normal range. In August 2025 (3 months after discontinuation), outpatient follow-up showed marked improvement in blood glucose, blood pressure (BP), body weight, and mobility: BP 120/70 mmHg, weight 64 kg, HbA1c 6.4%, fasting plasma glucose (FPG) 6.5 mmol/L. Antihypertensive and antidiabetic medications were reduced to bisoprolol fumarate 5 mg once daily and alogliptin benzoate 25 mg once daily. In October 2025 (5 months after discontinuation), follow-up at Ruijin Hospital, Shanghai Jiao Tong University School of Medicine showed FPG 6.5 mmol/L, 24 hours-UFC 166.25 μg/24 hours, ACTH < 1.5 pg/mL and morning serum cortisol (8 AM) 8.91 μg/dL. In January 2026 (8 months after discontinuation), follow-up at Shanghai Ruijin Hospital showed 24 hours-UFC: 200.79 μg/24 hours. In April 2026 (11 months after discontinuation), follow-up at Shanghai Ruijin Hospital, showed 24 hours-UFC: 216.90 μg/24 hours and ACTH < 1.5 pg/ml.

**Table 3:** Changes in 24 hours urinary free cortisol and adrenocorticotropic hormone during osilodrostat therapy

Time since the first dose (weeks)	24 hours urinary free cortisol (Μg/24 hours)	Adrenocorticotropic hormone (pg/mL)	Osilodrostat total daily dose (mg/d)
0	1068	0.09	2
2	1905	-	3
6	1836	-	4
8	1045	0.34	4
10	812	-	3.5
14	357.39	0.5	3
16	187.2	0.73	2.5
18	132.72	0.81	1.5
24	64.17	2.573	0
28	203.49	1.1	0
35	138.6	0.95	0
44	166.25	< 1.5	0
57	200.79	-	0
70	216.90	< 1.5	0

## Discussion

PBMAH is characterized by bilateral adrenal nodules larger than 1 cm and autonomous cortisol secretion. In 2013, studies demonstrated that the excess cortisol secretion in PBMAH is not driven by pituitary ACTH but by adrenal-derived ACTH acting in a paracrine manner [2]. The precise etiology of PBMAH has not been fully elucidated. Hypercortisolism in PBMAH is typically mediated by activation of aberrant G-protein-coupled receptors expressed in the adrenal cortex, which induce adrenal cell proliferation and excessive cortisol production. These aberrant receptors include those for gastric inhibitory polypeptide, β-adrenergic receptors, vasopressin receptors, 5-hydroxytryptamine receptor 4, luteinizing hormone/human chorionic gonadotropin receptor, and catecholamine-class hormone receptors. Upon binding to their ligands, these abnormal receptors activate intracellular signaling pathways, thereby promoting cortisol secretion [3, 4]. Currently, PBMAH is regarded as a hereditary disease. In 2013, Assié et al. thoroughly described ARMC5 gene mutations in PBMAH patients [5]. Reports have shown that germline inactivating mutations of the tumor suppressor gene ARMC5 account for approximately 20%–25% of sporadic PBMAH cases and 80% of familial cases [6]. At present, the molecular mechanisms of PBMAH remain incompletely understood and require further investigation.

The etiology, clinical manifestations, radiologic characteristics, and cortisol secretion patterns of PBMAH are highly heterogeneous. Early stages may be non-functional; however, as nodules enlarge, total cortisol secretion increases, and patients become clinically detectable due to hypercortisolism [5]. The present patient exhibited typical manifestations, including moon facies, buffalo hump, plethoric appearance, thin skin, and centripetal obesity. Laboratory and clinical evidence demonstrated metabolic disturbances induced by cortisol excess, including hyperglycemia, hypertension, and osteoporosis. Cortisol level was elevated, diurnal rhythm was disrupted, ACTH was suppressed, and both low-dose dexamethasone suppression tests (LDDST) and high-dose dexamethasone suppression tests (HDDST) were non-suppressible. Pituitary MRI revealed no pituitary adenoma. Adrenal CT showed bilateral diffuse enlargement with multiple low-density nodules. Genetic testing identified a pathogenic mutation in ARMC5, supporting the diagnosis of PBMAH, consistent with the clinical subtype.

Treatment of PBMAH includes surgical and pharmacological approaches and should be individualized. Most patients require surgical intervention, whereas those unable to tolerate surgery or with milder disease may receive medical therapy [7]. Bilateral adrenalectomy can effectively suppress excessive cortisol secretion [8]; however, lifelong glucocorticoid replacement is required after surgery, and patients may experience metabolic disturbances and an increased risk of adrenal crisis [9]. Therefore, unilateral total adrenalectomy has become the mainstream surgical strategy for PBMAH [10]. In the present case, the patient was unable to tolerate surgery, and at the time there were no approved pharmacologic treatments for CS in China (osilodrostat had not yet been marketed). However, her hypercortisolism required urgent management. After evaluation by the Department of Urology, left adrenal artery embolization (AAE) was performed. AAE was reported in the 1980s–1990s for adrenal cortical carcinoma and adrenal metastases with CS [11, 12], but the number of reported cases remains small, and its efficacy in CS is not well established. Currently, AAE is mainly used for adrenal tumors associated with primary aldosteronism. In this case, hypercortisolism did not improve following left AAE.

Osilodrostat is a potent 11β-hydroxylase (CYP11B1) inhibitor, approved in China in September 2024 for the treatment of adult CS, and is currently the only CS medication in China. But there are relatively few reports on its application in non-pituitary CS. In 2020, Tanaka T et al. reported its use in 9 patients with non-pituitary CS, with an overall remission rate of 77.8% at week 12 (7/9; 6 complete, 1 partial). Across patients with non-pituitary CS (including AIMAH, ectopic ACTH syndrome, and adrenal adenoma), osilodrostat produced a rapid and sustained reduction in mean urinary free cortisol (mUFC) [13]. A real-world study involving 103 patients with non-pituitary CS showed that after 12 weeks of osilodrostat therapy, 23 out of 45 patients (51.1%) in the osilodrostat intention to treat (ITT) population with non-CD CS achieved a complete mUFC response (mUFC ≤ upper limit of normal (ULN)) with non-missing values [14].

In the present case, 24 hours-UFC normalized after 16 weeks of osilodrostat, and the drug was discontinued after 23 weeks due to hypocortisolism, followed by short-term (7-day) hydrocortisone supplementation. Thereafter, without further osilodrostat or glucocorticoid replacement, repeated testing of morning serum cortisol and 24 hours-UFC remained within the normal range.

Notably, a reduction in the size of bilateral adrenal masses was observed during the period of osilodrostat therapy (Fig. 3). For imaging assessment, the maximum cross-sectional diameters were measured at the level of maximum adrenal dimension in the presence of diffuse enlargement [15], and the estimated maximum cross-sectional areas were calculated as the product of the orthogonal long and short diameters using the picture archiving and communication system (PACS) (Fig. 1, Fig. 3). After AAE, the left adrenal mass exhibited slight regression, while the right adrenal mass continued to enlarge, reflecting persistent hypercortisolism. A temporal association was observed between the biochemical control of cortisol levels and the subsequent reduction in bilateral adrenal mass dimensions during the period of osilodrostat treatment. However, it remains unclear whether the observed tumor shrinkage is attributable to osilodrostat, given that the patient had undergone prior AAE, which may have contributed to the morphological changes through ischemic mechanisms. Studies of AAE in primary aldosteronism have reported that no imaging changes in adrenal morphology were observed following the procedure [16, 17]; however, these data derive from a different disease context and may not be directly applicable to PBMAH. A literature review identified only two similar reports: in 2024, Sawabe F et al. described a patient with small-cell lung cancer and ectopic ACTH syndrome (EAS) who showed bilateral adrenal volume reduction after osilodrostat treatment [18]; and in 2026, Varlamov EV et al. found that in selected patients with ACTH-dependent CS, osilodrostat was associated with significant adrenal shrinkage [19].

**Fig. 4:**
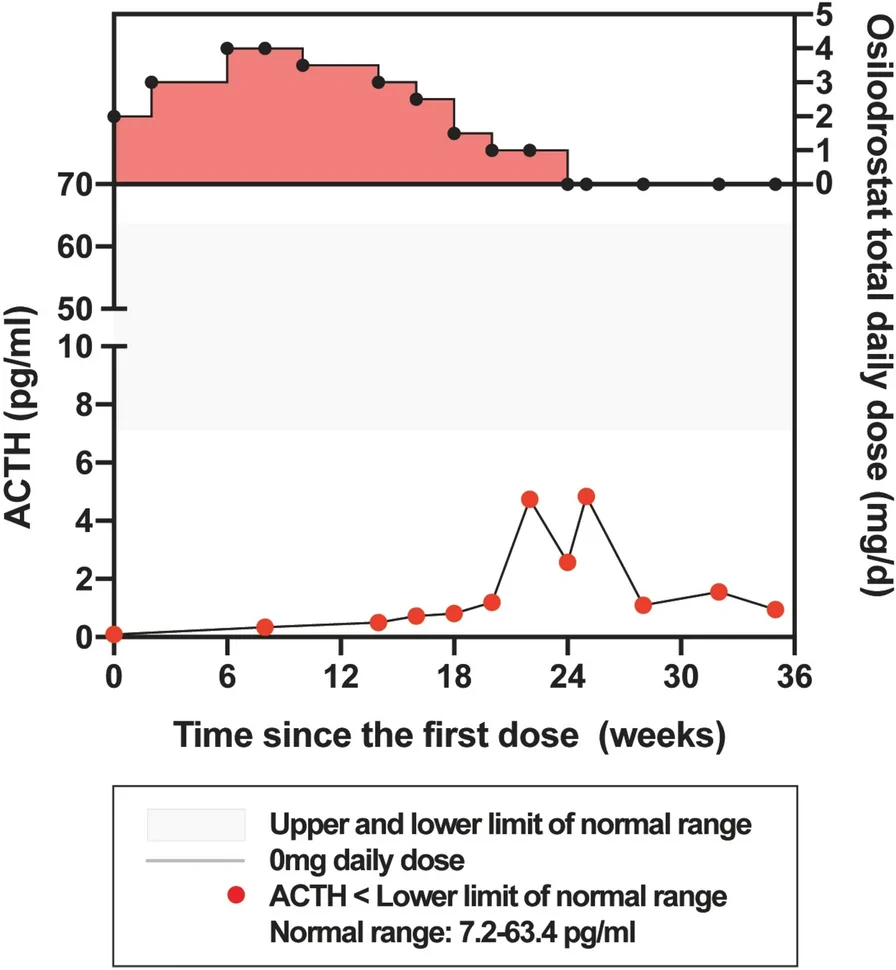
Changes in adrenocorticotropic hormone and corresponding dose adjustments during osilodrostat therapy

Osilodrostat reduces cortisol synthesis and secretion by inhibiting 11β-hydroxylase activity, thereby blocking conversion of 11-deoxycortisol to cortisol [20]. It is conceivable that prolonged inhibition of this key enzyme suppresses adrenal cortical cell function—particularly in hyperplastic cells—leading to markedly reduced synthetic activity. These cells may then undergo apoptosis or atrophy due to functional "inactivity", as if the drug forces highly active adrenal cells to "shut down", eventually resulting in atrophy or cell death. However, a review of the literature found no reports of adrenal shrinkage induced by ketoconazole or metyrapone—also 11β-hydroxylase inhibitors—suggesting that osilodrostat may be associated with this phenomenon through additional mechanisms. Notably, ACTH levels remained suppressed throughout the treatment period (Fig. 4, Table 3), even after cortisol normalization. The reasons for the lack of significant improvement in ACTH levels following osilodrostat treatment remain unclear and warrant further investigation.

The mechanism underlying the observed reduction in adrenal masses during the period of osilodrostat therapy remains unclear. However, during osilodrostat therapy in this patient, adrenal shrinkage, improvement in cortisol levels, and dose reduction appeared to progress in parallel, and clinical remission seemed linked to structural adrenal change. In 2021, a study by Wurth R et al. involving 44 PBMAH patients demonstrated that adrenal volume in patients carrying pathogenic ARMC5 variants positively correlated with 24-hour urinary 17-hydroxycorticosteroid (17-OHS) levels. When the left and right adrenal glands were analyzed separately, the correlation between left adrenal volume and 17-OHS was even stronger, and a significant correlation was also observed with urinary free cortisol (UFC) [21]. In our patient, improvement in cortisol and biochemical parameters may have been attributable to changes in adrenal volume. This suggests that radiologic monitoring of adrenal morphology during osilodrostat treatment may be warranted. If adrenal volume decreases, tapering or discontinuing osilodrostat—while maintaining adequate cortisol control—may be feasible.

## Conclusions

In summary, we report the first documented case of bilateral adrenal mass shrinkage observed in temporal association with osilodrostat treatment in a PBMAH patient. Treatment of PBMAH should be individualized; surgery remains the primary modality, while medical therapy may be applied in patients who cannot tolerate surgery or have milder disease. As the only CS medication currently approved in China, osilodrostat demonstrated favorable efficacy in this patient: after 23 weeks of therapy and 11 months of drug discontinuation, 24 hours-UFC remained within the normal range. Whether this patient can achieve sustained long-term remission warrants further observation.

This case is the first to report a reduction in bilateral adrenal masses observed following osilodrostat treatment in PBMAH. The finding suggests a potential association between drug therapy and the functional–structural regression of the adrenal glands, although the underlying mechanism remains unknown and causality cannot be established given the confounding effect of prior AAE. It indicates that osilodrostat may not only suppress cortisol synthesis, but its use may also be associated with changes in adrenal volume. The sustained remission after prolonged drug withdrawal in this patient may be linked to these structural changes, though the contribution of the preceding AAE cannot be excluded.

We recommend that in adrenal-origin CS treated with osilodrostat, radiologic monitoring of adrenal morphology should accompany biochemical evaluation in order to guide dose adjustment. At present, the mechanism underlying the reduction in adrenal volume observed in association with osilodrostat treatment remains unclear and requires further investigation.

## Data Availability

No datasets were generated or analyzed during the current study.

## Abbreviations

PBMAH: Primary bilateral macronodular adrenal hyperplasia
CS: Cushing's syndrome
ACTH: Adrenocorticotropic hormone
UFC: Urinary free cortisol
CLIA: Chemiluminescent immunoassay
AAE: Adrenal artery embolization
ARMC5: Armadillo repeat-containing protein 5
HPA: Hypothalamic–pituitary–adrenal
CYP11B1: Cytochrome P450 Family 11 Subfamily B Member 1
